# Accuracy of Teledentistry for Diagnosis and Treatment Planning of Pediatric Patients during COVID-19 Pandemic

**DOI:** 10.1155/2022/4147720

**Published:** 2022-11-19

**Authors:** Ozra Golsanamloo, Sanaz Iranizadeh, Amir Reza Jamei Khosroshahi, Leila Erfanparast, Ali Vafaei, Yalda Ahmadinia, Solmaz Maleki Dizaj

**Affiliations:** ^1^Department of Pediatric Dentistry, Faculty of Dentistry, Tabriz University of Medical Sciences, Tabriz, Iran; ^2^Faculty of Nursing and Midwifery, Tabriz University of Medical Sciences, Tabriz, Iran; ^3^Dental and Periodontal Research Center, Tabriz University of Medical Sciences, Tabriz, Iran

## Abstract

Teledentistry is a new technology in the dentistry field, which has great benefits during pandemic such as the coronavirus disease 2019 (COVID-19). The overall purpose of the study was to assess the diagnostic sensitivity and specificity of virtual (mobile phone teledentistry) compared with clinical examinations during COVID-19. The basic design of the study was based on the comparison treatment plans by the students and the gold standard (clinical treatment plan of an expert pedodontist with 10 years of clinical experience). This double-blind clinical trial was conducted on 20 children (aged 6 to 12 years) with a chief complaint of dental caries with or without pain. An appropriate radiograph and five standard intraoral photographs (frontal view occlusion, maxillary occlusal view, mandibular occlusal view, right lateral view, and left lateral view) were prescribed for each patient according to the guidelines of the American Association of Pediatric Dentistry. Then, the treatment plan for the carious teeth was recorded for each patient. Each patient underwent a clinical examination at first and was followed randomly by a virtual examination by two dental students. Then, the clinical and virtual treatment plans were compared with each other, and also with the gold standard. The sensitivity and specificity values were calculated for each group. The accuracy of the diagnosis was measured by applying Cohen's kappa. Interexaminer reliability was measured using the intraclass correlation coefficient (ICC) and Cronbach's alpha. The mean kappa coefficient for the interexaminer agreement (for 24 teeth) was 0.62 in clinical and 0.69 in virtual examinations. The results showed no significant difference in the treatment plans of students and the gold standard (*P* > 0.05). The diagnostic sensitivity and specificity were 73.22% and 95.8% for clinical and 76.44% and 92.9% for virtual treatment plans showing no significant differences between virtual (mobile phone teledentistry) and clinical examinations (*P* > 0.05). The intraexaminer reliability of the examiners was found to be 0.92 by calculating the ICC. Then, teledentistry can be considered as a supplement to clinical examinations of pediatric dentistry, finally resulting in better patient management. However, more studies are necessary for teledentistry.

## 1. Introduction

The coronavirus disease 2019 (COVID-19) pandemic has challenged the healthcare systems worldwide. It has impacted almost all countries and was declared a pandemic by the World Health Organization (WHO) [1]. Almost all dental procedures produce aerosols, which can cause virus transmission from the patients to dental staff and vice versa [2, 3]. Thus, many advisory and regulatory dental organizations worldwide have recommended utmost precautions in dental treatments during the COVID-19 pandemic and asked for provision of only emergency services during this period [1]. On January 30, 2020, WHO stated the occurrence of COVID-19 as a “public health emergency of international concern” [4–6]. As a consequence, the current health crisis has offered new challenges to dentists, motivating new priorities, such as ensuring patient safety, regulating and reducing contamination hazards, and enhancing the treatment efficacy to increase productivity and effectiveness. So, new approaches, apparatuses, and a more applications of digital health have occurred [7].

Considering the ascending trend of COVID-19 cases, this pandemic does not appear to end any time soon [1]. Given that the speculations in this respect are true, and COVID-19 becomes endemic, revision and innovation of dental care services would be required to continue service provision with minimal risk of infection. Teledentistry can serve as a new solution for continuing dental care during the COVID-19 pandemic and also in the future.

Teledentistry refers to the use of electronic information and telecommunication technology to support long-distance oral clinical care, professional health-related education, and is patient-related. Teledentistry can be applied as a supplement to clinical studies of paediatric dentistry, as a final point causing enhanced patient handling. This new technology can lead to an important contribution in decreasing the supply-demand gap of paediatric dental specialists in spaces where healthcare services are incomplete and guarantee safety throughout the pandemic at the same time as offering dental care to paediatric patients and providing good health management [3].

Known challenges of teledentistry included patient and clinician's acceptance to have acceptable infrastructure, reimbursement, and security concerns. Approaches for solving these worries included clinician and patient teaching and using Health Insurance Portability and Accountability Act-compliant requests. Providing care for patients during the pandemic and spreading care to spaces lacking access to dental care are the main benefits of teledentistry [8].

There are two telemedicine modes: real-time consultation with patients, and storing and forwarding the information. The latter modality provides excellent results for most dental procedures without excessive costs for the equipment or connectivity [9–12].

Telemedicine by using smartphones is a subset of telemedicine, which combines smartphone technology and telemedicine (storage and transfer of medical information) for oral care services. Although dental photography is now an inseparable part of the routine dental practice, it is rarely used for education, diagnosis, consultation, or referral in routine dental practice. The main subunits of teledentistry include telecounseling, teletriage, telemonitoring, and follow-up, and telediagnosis [9, 13]. Teledentistry can be used for long-distance clinical education of students as well [14]. Some clinical topics in dental curricula such as orthodontics and oral and maxillofacial radiology can be well taught virtually [15, 16]. In these topics, patient data can be collected and easily discussed with dental students by using teledentistry tools, even in the physical absence of patients [15]. Teledentistry can also help in problem-based learning with students without requiring their physical presence [17].

In view of prior studies, Steinmeier et al. conducted a cross-sectional validation study to assess the levels of agreement between remote diagnoses (derived from Intraoral scans (IOS)) and diagnoses based on clinical examinations for assessing dental and periodontal conditions. The remote examination using IOS was effective in detecting dental findings, whereas periodontal conditions could not be assessed with the same accuracy. According to the authors, the remote assessment of IOS would allow a time-efficient screening and triage of patients. Improvement of the image quality of IOS may further allow increase in the accuracy of remote assessments in dentistry [18]. Flores et al. conducted a study to assess the diagnostic accuracy of a telediagnosis service for oral mucosal disorders. Based on the results, telediagnosis for oral diseases can be considered a reliable method, representing a promising alternative for the clinical support of health professionals, particularly in remote locations [19]. AlShaya et al. tested the reliability of mobile phone teledentistry in the diagnosis and treatment planning of dental caries in children in mixed dentition. They concluded that although the use of teledentistry without radiographs is not as accurate as clinical examination, mobile phone teledentistry offers acceptable reliability for the initial diagnosis of caries in children [20].

To the best of the author's knowledge, no previous study has compared the diagnostic and treatment planning skills of undergraduate dental students in teledentistry and in the clinical setting.

Using of teledentistry in the education of undergraduate dental students could be useful in the following cases:

- Improving clinical judgment and diagnostic and treatment planning skills of undergraduate dental students
- Preparing students to use this method in their future careers and to use teleconsultation with specialists when needed
- Reducing the need for patients to visit the dentist in pandemics such as the COVID-19 pandemic
- Collaborating between colleagues

Thus, considering the current status of the COVID-19 pandemic, and the high risk of cross-contamination in dental practice, teledentistry can greatly help in the education of dental students. The present study aimed to assess the accuracy of teledentistry in diagnosis and treatment planning by undergraduate dental students in comparison with a clinical examination based on tooth type (primary and permanent) to compare the treatment plans by the students and the gold standard during the COVID-19 pandemic.

The main questions for this study were;

Is there any significant difference between virtual (mobile phone teledentistry) and clinical examinations based on tooth type (primary and permanent) using the treatment plans by the students during the COVID-19 pandemic?

Is there any significant differences between the treatment plans by the students and the gold standard during the COVID-19 pandemic?

## 2. Materials and Methods

This interventional study has been performed by 40 dental students involved with the treatment of 20 pediatric dental patients referred to the Pediatric Dentistry Department of the Faculty of Dentistry, Tabriz University of Medical Sciences in 2021. The children were between 6 to 12 years of age with a chief complaint of dental caries with or without pain. Children with systemic conditions affecting their treatment plan and those whose parents did not consent to their participation were excluded. Written informed consent was obtained from the parents regarding the participation of their children in the study.

The patients first underwent clinical oral examination by an expert pedodontist with 10 years of clinical experience (gold standard) and the necessary radiographs were requested for them. Also, five intraoral standard photographs were obtained from each patient according to the guidelines of the American Association of Pediatric Dentistry. The intraoral photographs were obtained by a high-quality mobile phone camera (iPhone 13, Apple Corp. Cupertino, CA).

The photographs were: (i) frontal view occlusion, (ii) maxillary occlusal view, (iii) mandibular occlusal view, (iv) right lateral view, and (v) left lateral view.

Then, the treatment plan for carious teeth was recorded according to the chart for registration of oral findings and the suggested treatment plan released by Indiana University. The treatment plans for carious teeth included restorative treatment, pulp therapy and restoration, and tooth extraction. Then 40 undergraduate dental students were instructed on how to fill out the chart.

All 40 dental students and 20 pediatric dental patients were coded, and each patient was randomly examined by two dental students independently. Clinical examinations were first performed face-to-face, and then virtually. In other words, each dental student performed both clinical and virtual examinations randomly. Finally, the clinical and virtual treatment plans designed by dental students were compared with each other and also with the gold standard.

It should be noted that since dental students have different levels of skills in diagnosis and treatment planning, the kappa coefficient of the agreement was first calculated for the examiners. Both dental students and pedodontists (gold standard) who performed both types of examinations were blinded to the process performed by another examiner (double-blind design).

## 3. Sample Size

The sample size was calculated according to a study by AlShaya et al., [20] assuming the diagnostic sensitivity and specificity of treatment planning to be 80% and 90%, respectively, alpha = 0.05. To increase the reliability of the results, the sample size was increased by 20%, and finally, 40 students were enrolled.

## 4. Validation of the Diagnosis

The accuracy of the diagnosis was measured by applying Cohen's kappa. Interexaminer reliability was measured using the intraclass correlation coefficient (ICC) and Cronbach's alpha.

## 5. Results

Twenty patients were evaluated in this study (8 males and 12 females). The mean age of the patients was 7.8 years. A total of 480 teeth (4 permanent teeth, and 20 primary teeth for each patient) were examined by 40 dental students. Each patient was examined by two students. As shown in Table 1, the mean kappa coefficient for interexaminer agreement (for 24 teeth) was 0.62 for clinical examination and 0.69 for virtual examination.

Table 2 Shows that the percentage of carious teeth as diagnosed by both examiners was not significantly different in clinical and virtual examinations (*P* > 0.05).

As indicated in Table 3, no significant difference was noted in the treatment plans of students and the gold standard (*P* > 0.05).

*P* value by the Chi-square test in comparison with that done by expert pedodontist.

Table 4 shows the diagnostic sensitivity and specificity of virtual and clinical examinations based on tooth type (primary and permanent). A comparison of the treatment plans by the students and the gold standard revealed that the sensitivity and specificity were 83.6% and 95.8%, respectively, in clinical examination. These values were 84.2% and 92.9%, respectively, in virtual examination.

The intraexaminer reliability was assessed by calculating the intraclass correlation coefficient (Table 5). The Cronbach's alpha was 0.92 for intraexaminer agreement.

## 6. Discussion

Teledentistry is a suitable method for regular systematic dental check-ups and examinations in children, adolescents, and adults that facilitate access to patients' dental status based on the electronic database and history of patients [9, 13].

In the present study, a total of 480 teeth of 6-12 years old children were examined by 40 dental students (each patient was examined by two dental students). The mean kappa coefficient (for 24 types of teeth) for the interexaminer agreement was found to be 0.62 for clinical and 0.69 for virtual examinations (Table 1). Also, the percentage of carious teeth as diagnosed by both examiners was not significantly different in clinical and virtual examinations (Table 2). In both clinical and virtual examinations, the treatment plans offered by dental students had no significant difference from the gold standard (Table 3). The diagnostic sensitivity and specificity were 73.22% and 95.8% for clinical and 76.44% and 92.9% for virtual treatment, respectively. The results also showed that the sensitivity and specificity were 80.07% and 94.20% for clinical examination of primary teeth while these values were 80.34% and 89.59% for virtual examination, respectively. The sensitivity and specificity for permanent teeth were 45.83% and 88.95% in clinical examination and 60.83% and 93.40% for virtual examination, respectively, (Table 4). The intraexaminer reliability was calculated as ICC = 0.92, which was favorable (Table 5).

AlShaya et al. [20] evaluated the reliability of mobile teledentistry for diagnosis and treatment planning in patients in the mixed dentition period. A total of 57 children between 6 to 12 years were examined by 6 pedodontists in the teledentistry group. The photographs sent by patients were assessed without dental radiographs. In their study, the diagnostic sensitivity was higher than specificity, although both values were over 80% in all cases. The reliability of teledentistry for primary teeth was higher than for permanent teeth in their study. They concluded that although the use of teledentistry without radiographs is not as accurate as clinical examination, mobile phone teledentistry offers acceptable reliability for the initial diagnosis of caries in children. In the present study children between 6-12 years examined who were in a mixed dentition period, enabling examination of both primary and permanent teeth by dental students. In this study, the sensitivity and specificity of primary teeth were higher than permanent teeth. Also, both values were over 80% in primary teeth. We also compared the treatment plans by the students and the gold standard.

Adlassnig et al. [21] reported a sensitivity of 94-100% and a specificity of 52-100% for teledentistry diagnosis compared to clinical examination. In their study, the diagnosis of dental problems based on noninvasive photographs and the examiners was different in noninvasive photography and clinical examination groups. In our double-blinded study, dental students randomly performed both clinical and virtual examinations. Thus, each patient was examined by two dental students by the two examination modes that may decrease observer bias.

Queyroux et al. [22] reported that the diagnostic sensitivity of photographs taken by smartphone cameras ranged from 60% to 68% while the diagnostic specificity ranged from 97% to 98%. In line with their findings, the present study showed higher diagnostic specificity than sensitivity although both values were within the acceptable range.

Subbalekshmi et al. [23] indicated that effective screening for early childhood caries was feasible by assessment of children's digital dental photographs taken at school, and paves the way for the application of teledentistry. In their study, Cronbach's alpha for interexaminer reliability was found to be 0.98 for the two modalities of clinical examination and digital photography by the camera, which indicates high reliability. In the present study, relatively low interexaminer reliability (kappa of 0.6) in both clinical and virtual examinations can be due to the low experience of dental students.

Purohit et al. [24] reported acceptable diagnostic accuracy of teledentistry such that it was comparable to clinical examination. They suggested that it can serve as an adjunct for dental caries screening, consultation, and oral health strategy planning for children. The above-mentioned studies were all in agreement with the present study and believed in the feasibility of teledentistry.

Kohara et al. [25] demonstrated that sound tooth surfaces can be precisely distinguished from extensively carious surfaces on photographs taken by smartphones. In their study, the authors compared two different models of smartphones and a camera with clinical examination for detection of different grades of carious lesions of primary molars in children between 3-6 years. In the virtual examination, two examiners who were blinded to the type of camera used for taking the photographs examined the photographs independently on a monitor screen. Next, in the clinical phase, the teeth were directly examined clinically by two other examiners (the results served as the standard reference). The kappa coefficient was calculated to be 0.7 for the agreement of photography with macrocamera and clinical examination, and 0.9 for the agreement of photography with iPhone and Nexus smartphones and clinical examination. In their study, similar to the present study, virtual and clinical examinations were performed by different clinicians. However, the present study had a different method in that each patient was examined by two dental students, and the results were compared with the diagnosis of a pedodontist with 10 years of clinical experience as the gold standard, which increases the accuracy of the results. Another finding of the present study was the comparison of treatment plans offered virtually and clinically, which showed no significant difference from the gold standard treatment plan. Finally, in the present study using Smartphone technology simply capture and store digital photographs and help dental clinicians to acquire diagnostic data with acceptable accuracy for teledentistry purposes.

## 7. Limitations and Recommendations

We recommend that stakeholders increase educational efforts about teledentistry among the general population and dental schools to raise awareness of teledentistry and increase acceptance of dental patients and improve clinical judgment and diagnostic and treatment planning skills of undergraduate dental students, as recommended in other studies [3]. The main limitation of this study was the small sample size, which was due to the decrease in the number of patients visiting the dentist due to the COVID-19 pandemic and future studies should also use larger sample sizes to provide more generalizable results.

## 8. Conclusion

The outcomes presented no significant difference in the treatment plans of students and the gold standard (*P* > 0.05). The diagnostic sensitivity and specificity were 73.22% and 95.8% for clinical and 76.44% and 92.9% for virtual treatment plans showing no significant differences between virtual (mobile phone teledentistry) and clinical examinations (*P* > 0.05). The intraexaminer reliability of the examiners was found to be 0.92 by calculating the ICC. Teledentistry can be a complement procedure to face-to-face clinical techniques of paediatric dentistry, leading to better patient management. This technology can make a significant contribution to reducing better patient management in places with limited healthcare facilities. This method can lead to safeguarding safety during the pandemic. It can provide safe dental care management to paediatric patients. Additional studies are necessary for harmless and evidence-based usage of teledentistry in the field of dentistry, especially paediatric dentistry.

## Figures and Tables

**Table 1:** Mean kappa coefficient for interexaminer agreement in clinical and virtual examinations (*n* = 480).

Interexaminer agreement	Clinical examination	Virtual examination
Mean kappa coefficient	0.62	0.69

**Table 2:** Comparison of the frequency of carious teeth detected clinically and virtually by the two dental students and the gold standard for 24 types of teeth.

Teeth type	Teeth number or code	Gold standard	First dental student			Second dental student		
		Clinical	Clinical	Virtual	*P* value	Clinical	Virtual	*P* value
Permanent teeth	14	25.00%	20.00%	25.00%	0.500	25.00%	25.00%	0.642
	19	55.00%	45.00%	35.00%	0.374	40.00%	55.00%	0.264
	3	65.00%	60.00%	55.00%	0.171	60.00%	60.00%	0.500
	30	10.00%	20.00%	5.00%	0.500	10.00%	15.00%	0.500

Primary teeth	A	20.00%	25.00%	20.00%	0.366	25.00%	20.00%	0.500
	B	30.00%	25.00%	35.00%	0.358	20.00%	25.00%	0.500
	C	25.00%	20.00%	30.00%	0.500	20.00%	25.00%	0.500
	D	20.00%	30.00%	25.00%	0.669	20.00%	25.00%	0.669
	E	20.00%	15.00%	15.00%	0.500	15.00%	15.00%	0.376
	F	55.00%	55.00%	50.00%	0.500	60.00%	50.00%	0.371
	G	75.00%	75.00%	70.00%	0.331	60.00%	70.00%	0.698
	H	15.00%	10.00%	20.00%	0.500	10.00%	10.00%	0.642
	I	25.00%	15.00%	20.00%	0.669	25.00%	25.00%	0.347
	J	85.00%	85.00%	85.00%	0.500	75.00%	85.00%	0.500
	K	85.00%	75.00%	70.00%	0.500	85.00%	90.00%	0.500
	L	15.00%	15.00%	10.00%	0.500	15.00%	10.00%	0.723
	M	0.00%	0.00%	0.00%		5.00%	5.00%	0.756
	N	0.00%	5.00%	5.00%	0.500	5.00%	5.00%	0.756
	O	0.00%	10.00%	5.00%	0.500	0.00%	0.00%	
	P	0.00%	0.00%	0.00%		5.00%	5.00%	0.756
	Q	10.00%	5.00%	15.00%	0.302	10.00%	15.00%	0.723
	R	95.00%	70.00%	85.00%	0.255	85.00%	85.00%	0.669
	S	95.00%	90.00%	80.00%	0.321	90.00%	90.00%	0.698
	T	30.00%	20.00%	15.00%	0.500	15.00%	20.00%	0.500

**Table 3:** Frequency distribution of treatment plans presented by the examiners in virtual and clinical examinations.

Treatment plan	Gold standard	Clinical examination		Virtual examination	
		First student	Second student	First student	Second student
No treatment required	309(64.38)	322 (67.08)	324 (67.50)	325 (67.71)	314 (65.42)
Restorative treatment	53(11.04)	65 (13.54)	68 (14.17)	58 (12.08)	68 (14.17)
Pulp therapy	67(13.96)	57 (11.88)	59 (12.29)	59 (12.29)	57 (11.88)
Extraction	51(10.63)	36 (7.50)	29 (6.04)	38 (7.92)	41 (8.54)
*P* value		0.759	0.534	0.638	0.732

**Table 4:** Sensitivity and specificity for detection of sound and carious teeth in virtual and clinical examinations.

Index	All teeth		Permanent teeth		Primary teeth	
	Clinical	Virtual	Clinical	Virtual	Clinical	Virtual
Sensitivity	73.22%	76.44%	45.83%	60.83%	80.07%	80.34%
Specificity	95.8%	92.9%	88.95%	93.40%	94.20%	89.59%

**Table 5:** Intraexaminer reliability by calculating the intraclass correlation coefficient for single measures and average measures.

Measure type	Intraclass correlation	95% confidence interval		*F* test with true value 0			
		Lower bound	Upper bound	Value	df1	df2	*P* value
Single measures	0.368	0.245	0.547	12.639	23	437	<0.001
Average measures	0.921	0.866	0.960	12.639	23	437	<0.001

## Data Availability

The raw data for this study can be shared at this time.
